# Supplementary material for: Hybrid achromatic microlenses with high numerical apertures and focusing efficiencies across the visible
Source: Nat Commun. 2023 May 30;14:3119. doi: 10.1038/s41467-023-38858-y (PMC10229656; doi:10.1038/s41467-023-38858-y)
Supplement: Supplementary file 1 — Supplementary Information [file 41467_2023_38858_MOESM1_ESM.pdf]

Supplementary Information for:

Hybrid Achromatic Microlenses with High Numerical Apertures and Focusing Efficiencies Across the Visible

Corey A. Richards[1,2,3], Christian R. Ocier[1,2,3], Dajie Xie[1,2,3], Haibo Gao[1,2,3], Taylor Robertson[4], Lynford L. Goddard[3,5,6], Rasmus E. Christiansen[7], David G. Cahill[1,2,8], and Paul V. Braun[1,2,3,8*]

[1]Department of Materials Science and Engineering, University of Illinois Urbana-Champaign, Urbana, IL, USA. [2]Materials Research Laboratory, University of Illinois Urbana-Champaign, Urbana, IL, USA. [3]Beckman Institute for Advanced Science and Technology, University of Illinois Urbana-Champaign, Urbana, IL, USA. [4]Ansys Lumerical, Vancouver, British Columbia, Canada. [5]Department of Electrical and Computer Engineering, University of Illinois Urbana-Champaign, Urbana, IL, USA. [6]Holonyak Micro and Nanotechnology Laboratory, University of Illinois Urbana-Champaign, Urbana, IL, USA. [7]Department of Civil and Mechanical Engineering, Technical University of Denmark, Kongens Lyngby, Denmark. [8]Department of Mechanical Science and Engineering, University of Illinois Urbana-Champaign, Urbana, IL, USA. *email: pbraun@illinois.edu

Section 1: SCRIBE Lithography

The hybrid doublets presented in the main text are fabricated using our recently developed subsurface microscale 3D printing process, coined Subsurface Controllable Refractive Index via Beam Exposure (SCRIBE). Using SCRIBE, a nonlinear negative tone photoresist is soaked into the pores of porous silicon (PSi) or porous silicon oxide ($PSiO_2$) host mediums and undergoes nonlinear polymerization induced by a femtosecond pulsed laser. As shown in our previous works[1,2], the PSi and $PSiO_2$ have high transparency at the printing laser's wavelength (780 nm). For this work, only $PSiO_2$ was used because it has lower absorption than PSi at visible wavelengths.

Figure S1 outlines the fabrication process for producing SCRIBE hybrid lenses. PSi films are produced by electrochemically etching bulk silicon under an ethanolic hydrofluoric acid electrolyte. Pore formation is driven in the vertical direction, resulting in an anisotropic film. The films are then detached by electropolishing, and solvent transferred onto fused silica substrates. The PSi films stick strongly to their adopted substrates upon drying. PSi is converted to $PSiO_2$ by thermal oxidation, greatly reducing its anisotropy and refractive index[3,4]. Subsequently, the liquid photoresist is dripped onto the hosts and allowed to soak into the pores for 30 minutes. A femtosecond pulsed laser is focused into the volume of a $PSiO_2$ film, inducing multiphoton polymerization at the focal spot of the laser. The laser is scanned in the desired volumetric pattern to form the lenses. Unpolymerized photoresist is removed from the pores by developing in propylene glycol methyl ether acetate at 80°C. The samples are supercritically dried in liquid CO_2 to avoid cracking and pore collapse upon drying.

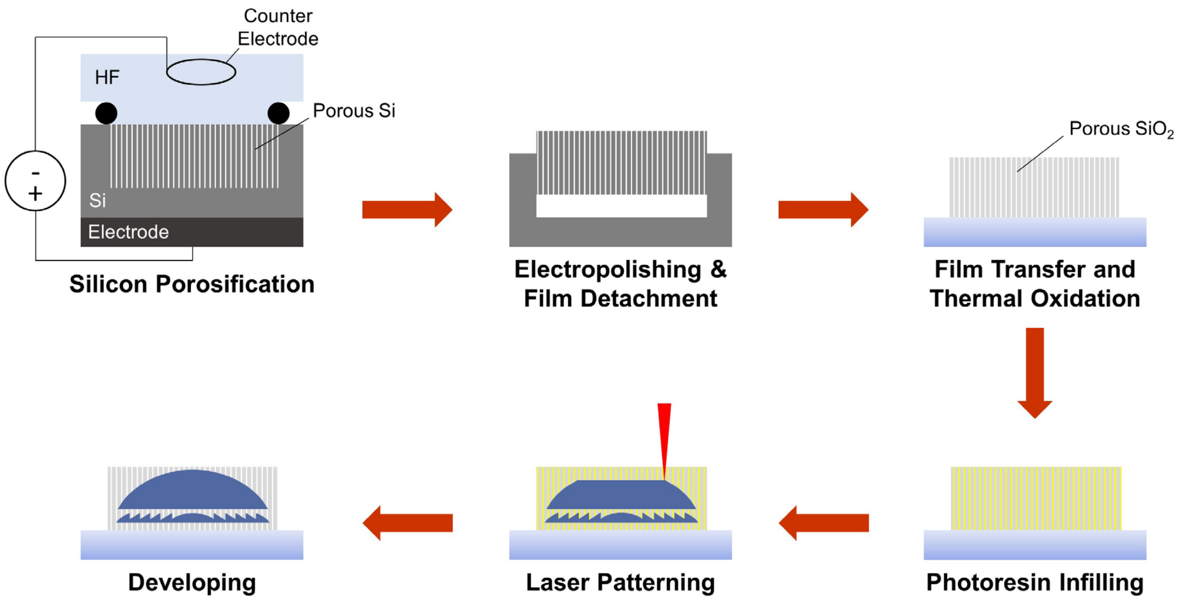

Fig. S1 | Schematic of the SCRIBE fabrication process.

Section 2: Measurement of Optimal Diffractive Lens Thickness

The thickness profile T(r) of a diffractive lens is given by the equation:

$$T(r) = T_{opt} * \left(\frac{\frac{2\pi}{\lambda} * \left(f - (f^2 + r^2)^{\frac{1}{2}} \right) + 2m\pi}{2\pi} + 1 \right) \qquad (S1)$$

where

$$T_{opt} = \frac{\lambda}{n_{lens} - n_{background}} \qquad (S2)$$

Here, T_{opt} is the optimal thickness over which a 2π phase shift occurs. It is challenging to measure the refractive index of a subsurface object. T_{opt} is estimated to be roughly 1.6 μm based on the data collected in our previous work[1]. To directly measure T_{opt}, we printed a series of diffractive lenses with identical zone widths as in Fig. 1d of the main text. The lenses were printed with different thicknesses in 100 nm intervals and the focusing efficiency of each lens was measured at 633 nm. Focusing efficiency vs diffractive lens thickness is plotted in Fig. S2. The highest focusing efficiency was measured when the diffractive lens thickness was 1.7 μm, so all diffractive lenses and hybrid components in this work were fabricated with this thickness.

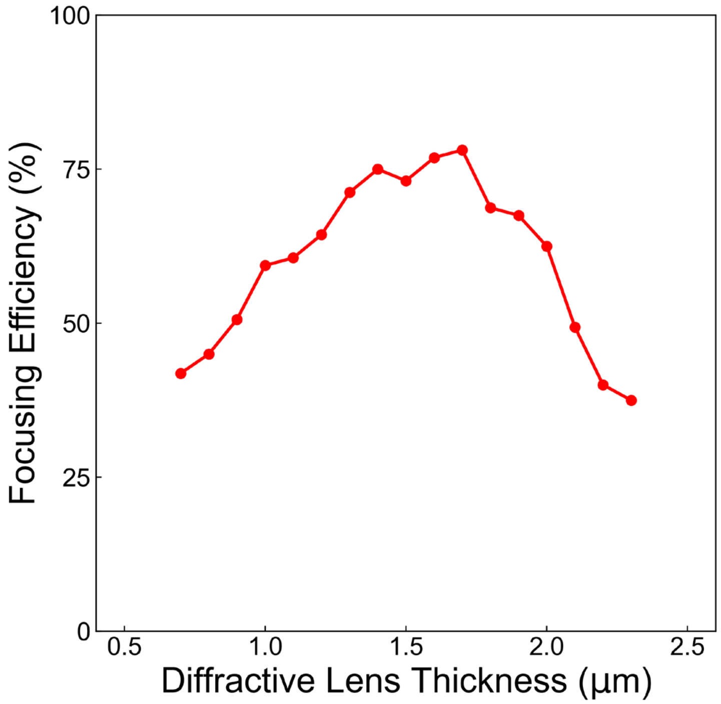

Fig. S2 | Focusing efficiency at 633 nm vs diffractive lens thickness. A series of diffractive lenses (from Fig. 1d in the main text) with different thicknesses was printed. The 1.7 μm thick lens had the highest focusing efficiency. Therefore, 1.7 μm is the optimal diffractive lens thickness for our materials system.

Section 3: GRIN Hybrid Lens Simulations

The refractive components of the GRIN hybrid doublets were formed by varying the laser power during fabrication, as describe in the Methods Section of the main text. The refractive GRIN components have a center-to-edge index contrast between 0.35 and 0.4 (the background index of the $PSiO_2$ host is around 1.1, the index of the edge of the GRIN component is slightly higher than 1.1, and the index of the center of the GRIN component is within the range of 1.48-1.52), as confirmed by the simulations in shown in Fig. S3. For these simulations, GRIN hybrid lenses were designed in COMSOL such that each GRIN refractive component has a different center-to-edge index contrast. The experimentally measured focal lengths most closely agree with simulation when the simulated center-to-edge index contrast is within the range of 0.35-0.4.

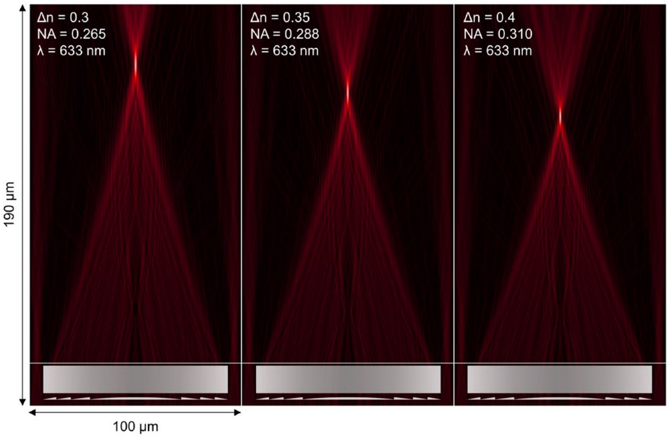

Fig. S3 | GRIN hybrid lens simulations. a, GRIN hybrid lenses were simulated in COMSOL where each refractive component has a different center-to-edge index contrast. An index contrast between 0.35 and 0.4 most accurately matches the average focal length of the experimentally fabricated lenses.

Section 4: Focal Spot Characterization

A confocal microscope captured X-Y images of the focal spots at the planes of maximum intensity for each lens and each wavelength. Figure S4 highlights the measured focal spots of a lower NA diffractive lens, a higher NA diffractive lens, a geometric hybrid doublet, and a gradient index (GRIN) hybrid doublet at 633, 612, 542, and 488 nm.

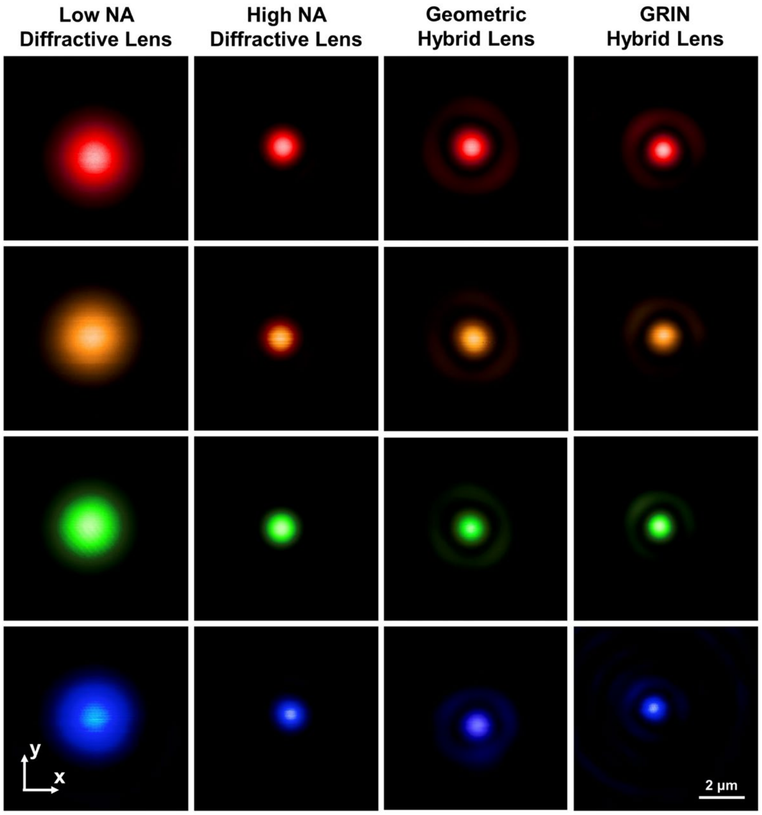

Fig. S4 | Focal plane scans. X-Y images were captured at the focal planes of each lens illuminated by 633 (red), 612 (orange), 542 (green), and 488 (blue) nm light. The focal spots of a single lower NA diffractive lens (first column), higher NA diffractive lens (second column), geometric hybrid lens (third column), and GRIN hybrid lens (fourth column) were measured.

The focal spots scans were used to estimate the lenses' Strehl ratios (SR). A single lens of each type was selected for the SR measurement. The collected data was smoothed for noise reduction and the normalized intensity was compared to that of an ideal lens of the same NA. SRs were measured over an area of $16\pi\lambda^2$, except in the case of the low NA diffractive lens which was measured within an area of $64\pi\lambda^2$ due to its larger spot size. The intensity profiles of the geometric hybrid doublet's focal spots are plotted above each intensity scan in Fig. S5a. The black curve is that of the ideal lens whereas the red curve is the normalized measurement. The SRs of each lens are plotted in Fig. S5b. While the low NA diffractive lens achieved diffraction limited focusing (SR $\geq$ 0.8), the SRs of the hybrid lenses are reduced due to fabrication errors. It is possible that we underestimated the SRs because we could not completely filter the background noise from the measurements. Additional details can be found in the Methods Section of the main text.

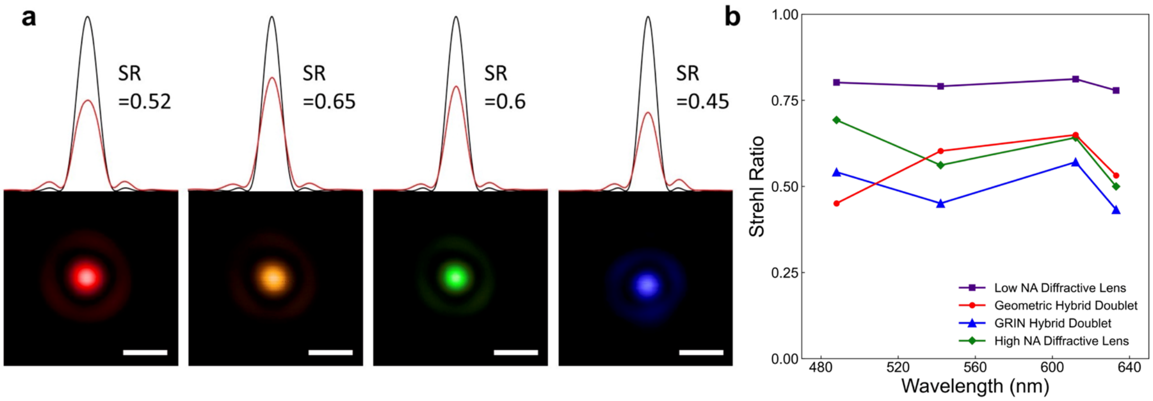

Fig. S5 | Strehl ratio measurements. a, X-Y scans of the focal planes of a geometric hybrid lens focusing 633, 612, 542, and 488 nm light. The normalized intensities of linecuts through the centers of each focal spot are displayed above the X-Y scans in red and compared to the normalized linecuts of ideal lenses of the same NAs in black. The Strehl ratio is the ratio between the maximum intensities of the measured and ideal linecuts. Scale bars are 2 µm. **b,** Plot of Strehl ratio vs wavelength for a lower NA diffractive lens, a higher NA diffractive lens, a geometric hybrid lens, and a GRIN hybrid lens. Each curve is from a randomly selected lens among the ten printed lenses of each type.

Section 5: Lens NAs

The focal lengths averaged between 10 fabricated microlenses were converted to NA (Fig. S6) according to:

$$NA = n * \sin\left(arctan\left(\frac{D}{2f}\right)\right) \qquad (S3)$$

where n is the refractive index of air, D is the lens diameter, and f is the focal length.

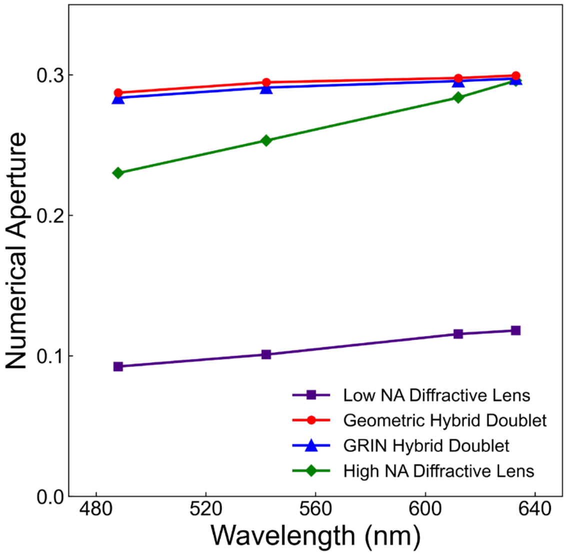

Fig. S6 | Average NA measurements. Graph of numerical aperture plotted against wavelength for the lower NA diffractive lenses, higher NA diffractive lenses, geometric hybrid lenses, and GRIN hybrid lenses. Each data point is averaged between 10 samples.

Section 6: Focusing Efficiency Simulations

Lens focusing efficiencies were simulated using the finite-difference time-domain (FDTD) method with Lumerical software. The details are available in the Methods Section of the main text. The simulated values are plotted in Fig. S7.

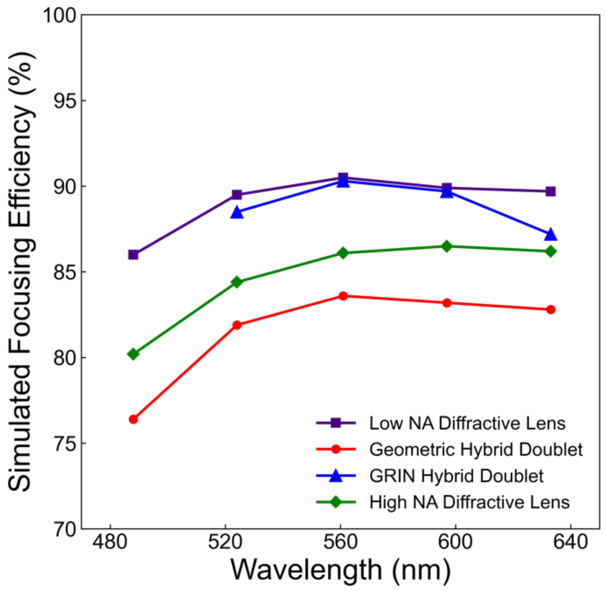

Fig. S7 | Simulated focusing efficiencies of diffractive and hybrid lenses using FDTD.

Section 7: Experimental Setup for Imaging with Microlenses

Imaging was performed using the setup in Fig. S8.

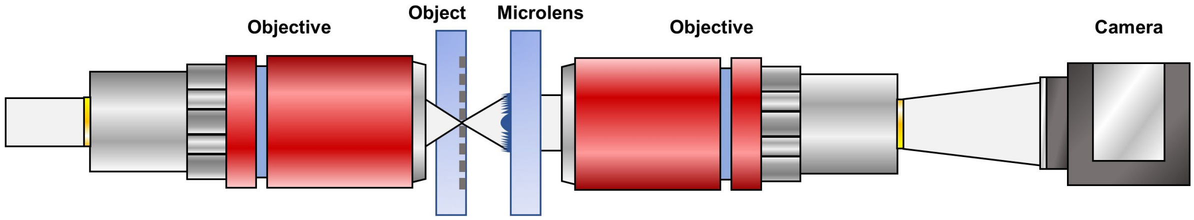

Fig. S8 | Experimental setup for imaging with microlenses. Experimental setup for forming white light images using microlenses. Light from a warm white LED with wavelength range 400-700 nm was collimated and focused onto the United States Air Force resolution target using an objective. The microlens collected the light and formed a real image of the target. The first objective and object were placed on a separated translating stage, allowing them to be moved towards and away from the microlens substrate. The distance between the target and the microlens was adjusted to roughly one focal length such that a real image was formed by the microlenses. The image formed by the microlens was collected by a second objective, which further magnified and projected the image onto a charge-coupled device.

The white light emitted by the LED experiences some dispersion caused by the collimation lens (Fig. S9). This negatively affected the quality of some images formed by the microlenses.

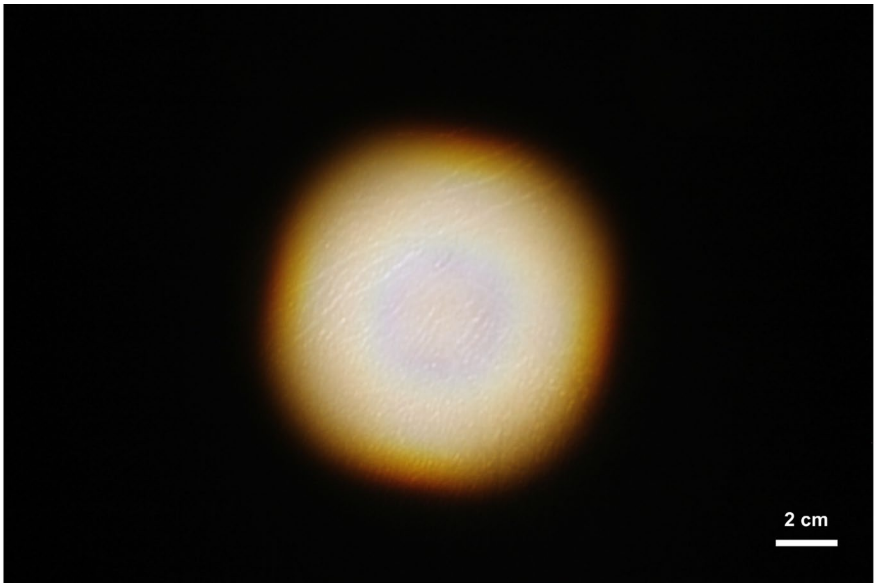

Fig. S9 | Dispersion of the white light LED caused by the collimation lens. Image of the collimated LED light shone onto a white background. Blue and purple fringing can be observed towards the center of the spot.

Reference images were taken of the resolution target direction with a 50x 0.42 NA objective (Fig. S10). Due to the dispersive collimation lens, the captured image exhibits some chromatic aberrations along the top and bottom of the imaged features. Similar aberrations can be seen in some of the broadband images formed by the microlenses.

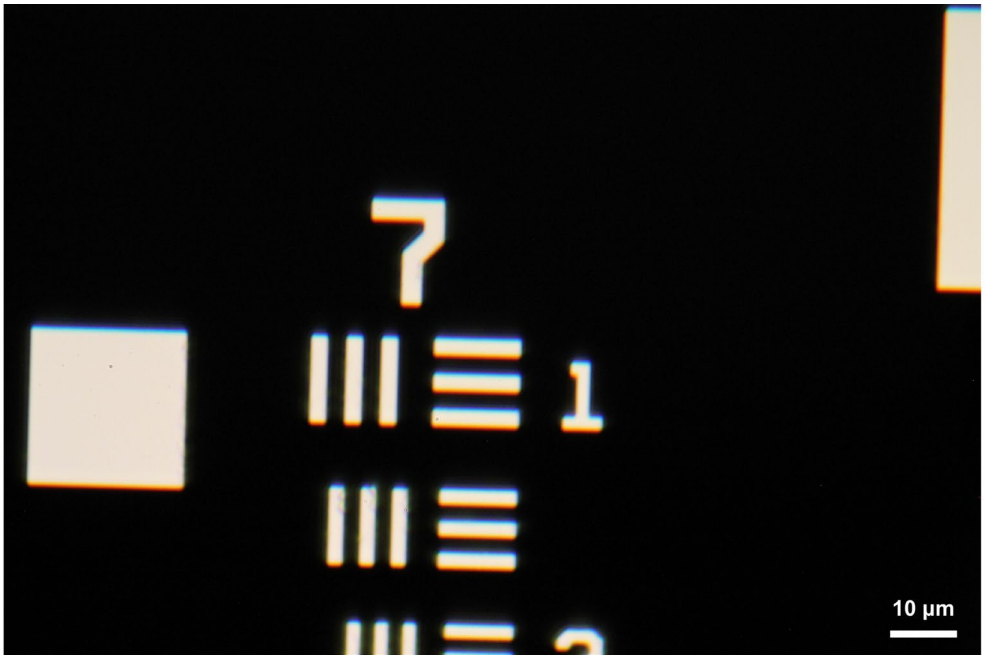

Fig. S10 | Reference image captured directly with an objective. Image of group 7 on the resolution target captured directly with an objective. Chromatic aberrations can be observed at the bottom and top of each feature.

Section 8: Additional Information on High NA Hybrid Microlenses

Additional information on the microlenses displayed in Fig. 5a-h in the main text is provided by Table S1. While the lens shown in Fig. 5a was averaged between 10 identical printed lenses, only one of each lens was measured for Figs. 5b-h.

Table S1: High NA Hybrid Microlens Measurements

Sample	NA at 633 nm	Focusing Error	Focusing Efficiency
Fig. 5a	0.3	2.8%	**488 nm:** 45.3% (avg.)<br>**542 nm:** 53.1% (avg.)<br>**612 nm:** 60% (avg.)<br>**633 nm:** 64% (avg.)
Fig. 5b	0.315	5%	**488 nm:** 42.3%<br>**542 nm:** 40%<br>**612 nm:** 40%<br>**633 nm:** 41.7%
Fig. 5c	0.347	3.6%	**488 nm:** 53.3%<br>**542 nm:** 45.5%<br>**612 nm:** 50%<br>**633 nm:** 37.8%
Fig. 5d	0.367	5%	**488 nm:** 50%<br>**542 nm:** 43.6%<br>**612 nm:** 42.6%<br>**633 nm:** 47.5%
Fig. 5e	0.375	5%	**488 nm:** 34.5%<br>**542 nm:** 37%<br>**612 nm:** 38.5%<br>**633 nm:** 33.5%
Fig. 5f	0.406	5.3%	**488 nm:** 38.5%<br>**542 nm:** 45%<br>**612 nm:** 41.7%<br>**633 nm:** 36.1%
Fig. 5g	0.46	7.3%	**488 nm:** 33.3%<br>**542 nm:** 30%<br>**612 nm:** 28.1%<br>**633 nm:** 21.7%
Fig. 5h	0.471	7.5%	**488 nm:** 38.9%<br>**542 nm:** 29.3%<br>**612 nm:** 35.7%<br>**633 nm:** 35%

Example X-Z scans of a hybrid achromat with NA=0.471 focusing 633, 612, 542, and 488 nm light is shown in Fig. S11.

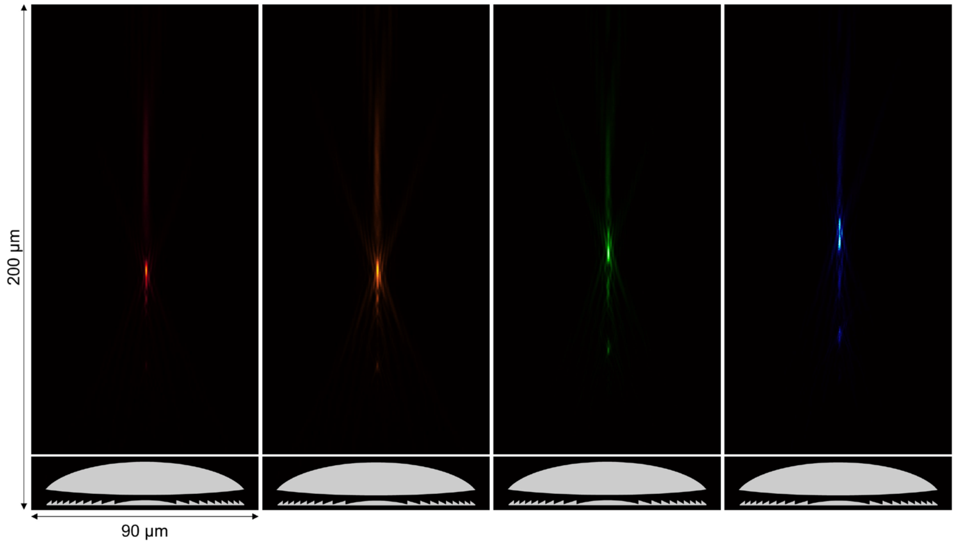

Fig. S11 | Focal profiles of a hybrid lens with an NA of 0.471 focusing light at visible wavelengths. A hybrid doublet is used to focus visible wavelengths with low dispersion and a high NA of 0.471

Section 9: Additional Information on Imaging with Achromatic Microlens Array

The amount of light-field information captured by the microlens array (MLA) is controlled by adjusting its distance from the resolution target. There is a tradeoff between amount of collected light-field information and image quality, as highlighted by Fig. S12. The images produced by the microlens array in Fig. S12a are sharper and higher magnification than those in Fig. S12b but contain less light-field information. The reconstructed images of Figs. S12a and S12b are shown in Figs. S12c and S12d, respectively. Due to a lack of collected light-field information, a full reconstruction cannot be accomplished using the image array in Fig. S12a, as seen in Fig. S12c. Conversely, the image array in Fig. S12b can be easily reconstructed, albeit with somewhat low quality (Fig. S12d). The image array in Fig. 6 of the main text finds a middle ground between image quality and light-field collection, resulting in a final reconstruction that is both complete and high quality.

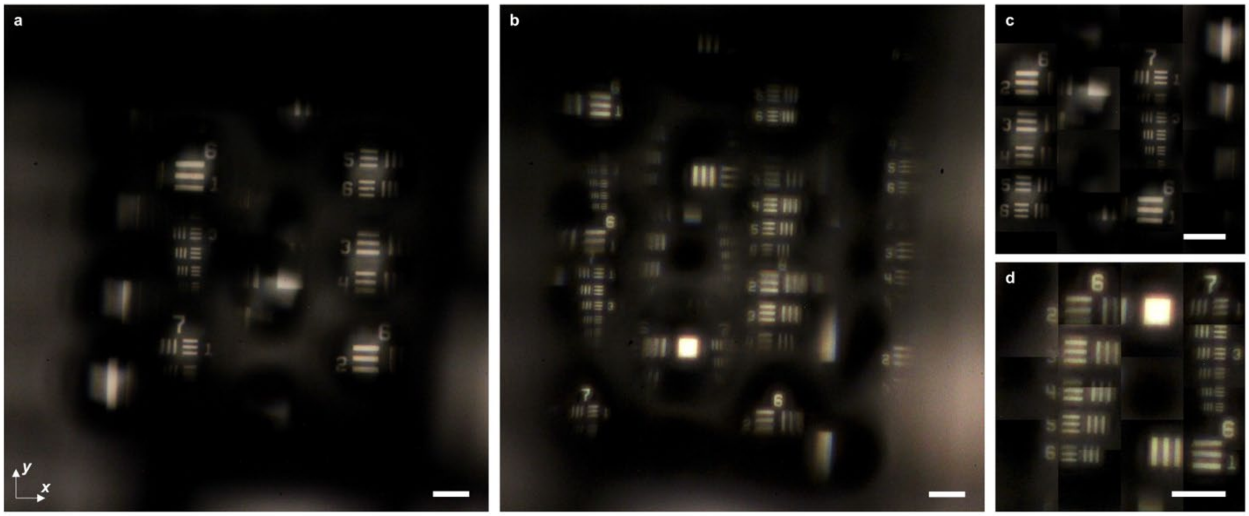

Fig. S12 | Achromatic microlens image arrays. a,b, Arrays of images formed by the microlens array in **Fig. 6a** of the main text under broadband white light illumination. The distance between the resolution target and the microlens array in **a** is just over one focal length. The distance between the resolution target and the microlens array in **b** is increased to collect as much light field information as possible while retaining relatively high image sharpness. Larger distance between the target and the microlens array enables the collection of more light-field information, but results in lower quality images. Light-field information of groups 6 and 7 on the USAF resolution target is collected. **c,d,** Images reconstructed from the image arrays in **a** and **b**, respectively. Scale bars are 45 μm.

Section 10: Comparison to Achromatic Metalenses in the Literature

A comparison between the achromatic hybrid lenses presented in this work and achromatic metalenses in the literature is presented in Table S2. Achromatic lenses are compared in terms of wavelength range, diameter, NA, focusing efficiency, and focal length error.

Table S2: Optical Performance of Different Achromatic Lens Designs

Ref.	Ref. in Main Text	Approach	λ Range (nm)	Diameter (μm)	NA	Focusing Efficiency (%)	Focal Length Error (%)
[5]	[3]	Library of Si pillars with complex geometries, generation 1A *	1300-1650	100	0.24	~45-56% †	Estimated by authors as 2-5%
[5]	[3]	Library of Si pillars with complex geometries, generation 1B *	1200-1650	100	0.24	~21.5-50% †	Estimated by authors as 2-5%
[5]	[3]	Library of Si pillars with complex geometries, generation 1B *	1200-1650	200	0.13	~21-56% †	Estimated by authors as 2-5%
[6]	[4]	Library of TiO_2 nanofins, geometric phase **	470-670	20	0.2	Stated as 20% at λ=500 nm by authors in the abstract	~3% for n=0 †
[6]	[4]	Library of TiO_2 nanofins, geometric phase **	470-670	220	0.02	~3-20% †	Not reported
[7]	[5]	Library of TiO_2 pillars with complex geometries *	650-1000	30	0.24	65-85%	Estimated by authors as <7%
[7]	[5]	Library of TiO_2 pillars with complex geometries *	650-1000	25	0.1	85-90.2%	Estimated by authors as <7%
[8]	[6]	TiO_2 fishnet metasurface *	640-1200	10, 15, and 20	~0.12, 0.09, 0.07 †	65-75%	Difficult to estimate from figure, not directly reported
[9]	[7]	Si pillars, computational wavefront coding *	1300-1700	490	0.238	~21-32% †	Difficult to estimate from figure, not directly reported
[10]	[8]	3D printing polymer pillars on phase plates *	1000-1800	20	0.27	39-78%	6.8%
[10]	[8]	3D printing polymer pillars on phase plates *	1000-1800	40	0.11	40-83%	4.5%
[10]	[8]	3D printing polymer pillars on phase plates *	1000-1800	80	0.06	48-75%	3.4%
[10]	[8]	3D printing polymer pillars and phase plates with air spacer *	1000-1800	40	0.32	18-34%	5.4%
[10]	[8]	3D printing polymer pillars and phase plates with air spacer *	1000-1800	100	0.24	13-22%	4.8%

[11]	[9]	Deep neural network, 1D simulation only	1310-1550	37	0.26	~33-75% †	~8% maximum from given reference focal length †
[11]	[9]	Deep neural network, 1D simulation only	1400-1650	24.6	0.24	~52-85% †	~5% maximum from given reference focal length †
[12]	[10]	GaN nanopillars and nanoholes **	400-660	50-60 †	0.106, 0.125, 0.15	~30-67%, 15-44%, 20-50% †	~6.4%, 9.2%, 11% maximum from given reference focal length †
[13]	[11]	TiO_2 nanofins *	460-700	26.4	0.2	~30% for white light † (reported vs polarization angle, not vs λ)	Stated as 9% by authors in the abstract
[14]	[12]	3D printing polymer nanoholes on phase plates *	450-1700	20	0.27	~35-80% †	Estimated by authors as 6%
[15]	[13]	Artificial neural net, TiO_2 pillars with complex geometries *	420-640	20.8	0.084	~10-84% †	~13% maximum from given central focal length †
[16]	[14]	TiO_2 nanofins **	488, 532, 658	2 mm	0.7, 0.3	12-16%, 10-12%	0.1%
[17]	[15]	Gradient-based optimization *	488, 532, 658	2 mm	0.7	15%	0.15%
[17]	[15]	Gradient-based optimization *	490, 520, 540, 570, 610, 650	2 mm	0.3, 0.7	8%	0.02%
[17]	[15]	Gradient-based optimization *	488, 532, 658	1 cm	0.3	15%	0.03%
This work	This work	Subsurface 3D printed Fresnel lens *	488-633	~90	0.3	~50%	12%
This work	This work	Subsurface stacked 3D printed hybrid lens, version 1 *	488-633	~90	0.3	51-70% maximum	<3%
This work	This work	Subsurface stacked 3D printed hybrid lens, version 2 *	488-633	~90	0.47	29-39%	7.5%

† = estimated values from a figure in the reference
* = polarization insensitive
** = polarization sensitive

References

1. Ocier, C. R. *et al.* Direct laser writing of volumetric gradient index lenses and waveguides. *Light: Science & Applications* **9**, 196 (2020).
2. Richards, C. A., Ocier, C. R., Zhu, J., Goddard, L. L. & Braun, P. V. Toward the realization of subsurface volumetric integrated optical systems. *Appl. Phys. Lett.* **119**, 130503 (2021).
3. Ocier, C. R., Krueger, N. A., Zhou, W. & Braun, P. V. Tunable Visibly Transparent Optics Derived from Porous Silicon. *ACS Photonics* **4**, 909–914 (2017).
4. Ocier, C. R. *et al.* Optically anisotropic porous silicon microlenses with tunable refractive indexes and birefringence profiles. *Opt. Mater. Express* **10**, 868 (2020).
5. Shrestha, S., Overvig, A. C., Lu, M., Stein, A. & Yu, N. Broadband achromatic dielectric metalenses. *Light: Science & Applications* **7**, 85 (2018).
6. Chen, W. T. *et al.* A broadband achromatic metalens for focusing and imaging in the visible. *Nature Nanotechnology* **13**, 220–226 (2018).
7. Wang, Y. *et al.* High-efficiency broadband achromatic metalens for near-IR biological imaging window. *Nat Commun* **12**, 5560 (2021).
8. Ndao, A. *et al.* Octave bandwidth photonic fishnet-achromatic-metalens. *Nature Communications* **11**, 3205 (2020).
9. Sun, T. *et al.* Polarization-insensitive achromatic metalens based on computational wavefront coding. *Opt. Express, OE* **29**, 31902–31914 (2021).
10. Balli, F., Sultan, M., Lami, S. K. & Hastings, J. T. A hybrid achromatic metalens. *Nature Communications* **11**, 3892 (2020).
11. An, X. *et al.* Broadband achromatic metalens design based on deep neural networks. *Opt. Lett., OL* **46**, 3881–3884 (2021).

12. Wang, S. *et al.* A broadband achromatic metalens in the visible. *Nature Nanotechnology* **13**, 227–232 (2018).

13. Chen, W. T., Zhu, A. Y., Sisler, J., Bharwani, Z. & Capasso, F. A broadband achromatic polarization-insensitive metalens consisting of anisotropic nanostructures. *Nature Communications* **10**, 355 (2019).

14. Balli, F., Sultan, M. A., Ozdemir, A. & Hastings, J. T. An ultrabroadband 3D achromatic metalens. *Nanophotonics* **10**, 1259–1264 (2021).

15. Wang, F. *et al.* Visible Achromatic Metalens Design Based on Artificial Neural Network. *Advanced Optical Materials* **10**, 2101842 (2022).

16. Li, Z. *et al.* Meta-optics achieves RGB-achromatic focusing for virtual reality. *Sci. Adv.* **7**, (2021).

17. Li, Z. *et al.* Inverse design enables large-scale high-performance meta-optics reshaping virtual reality. *Nat Commun* **13**, 2409 (2022).
